# Human Papillomavirus Infection and Cervical Neoplasia among Migrant Women Living in Italy

**DOI:** 10.3389/fonc.2014.00031

**Published:** 2014-02-20

**Authors:** Maria Lina Tornesello, Paolo Giorgi Rossi, Luigi Buonaguro, Franco Maria Buonaguro, HPV Prevalence Italian Working Group

**Affiliations:** ^1^Molecular Biology and Viral Oncology Unit, Istituto Nazionale Tumori “Fondazione G Pascale” – IRCCS, Naples, Italy; ^2^Servizio Interaziendale di Epidemiologia, Azienda Unità Sanitaria Locale and IRCCS, Arcispedale Santa Maria Nuova, Reggio Emilia, Italy

**Keywords:** human papillomaviruses, migrants, cervical cancer, Italy, cervical cancer screening

## Abstract

Human papillomavirus (HPV) infection is highly prevalent in women migrating from countries where cervical screening is not implemented. The variety of HPV genotypes, their prevalence and the association with cervical abnormalities has been investigated by several groups in women moving mainly from Eastern Europe, Africa, and Southern Asia to Italy. All studies are concordant on the elevated rate of HPV infection among immigrants, which is four times higher than that observed among age-matched Italian women. The HPV prevalence among short-term migrants and characterization of viral variants showed that the high prevalence of HPV reflects either individual lifestyle or high prevalence of HPV in the country of origin. The high burden of HPV infection correlates very well with the high incidence of cervical cancer in migrant women. In fact, during the years 2000–2004 the cervical cancer incidence in women from Central and Eastern Europe and living in Central Italy was 38.3 per 100,000, which is statistically significant higher than that of native Italian women (6 per 100,000). In this study, we pooled together the results of three independent studies originally designed to assess the distribution and the prevalence of HPV genotypes among 499 immigrant women living in Southern Italy. A total of 39 mucosal HPV genotypes were identified. The 12 genotypes (HPV16, 18, 31, 33, 35, 39, 45, 51, 52, 56, 58, and 59) classified as carcinogenic to humans (group 1) accounted for >80% of all infections. HPV16 was the most common viral type in all groups with frequency rates ranging from 15.4% in Africa to 51.1% in Eastern and Southern European HPV-positive women. The high prevalence of oncogenic HPVs and cervical cancer risk among migrant women, together with the lower participation in screening programs, demands for an urgent implementation of preventive strategies to increase screening and vaccine coverage and viral monitoring of uncommon HPV genotypes potential spreading in settled population.

## Introduction

Cervical cancer is the third most common cancer in women worldwide with an estimated 530,000 new cases in 2008 (1). More than 85% of the global burden occurs in developing countries, including Eastern, Western, and Middle Africa, Central America, South-Central Asia, and Melanesia, where it is the leading cause of cancer-related death among women (2). The etiological role of human papillomaviruses (HPVs) in cervical cancer was first proposed by Harald zur Hausen and his group in early 1970s (3). Nowadays more than 50 HPV genotypes have been found to infect the anogenital mucosa and most of them have been classified by the International Agency for Research on Cancer (IARC) as group 1 “high-risk” viruses (HPV16, 18, 31, 33, 35, 39, 45, 51,52, 56, 58, and 59), the 12 most frequently identified in cervical cancer; as group 2A “probably carcinogenic” (HPV68) and group 2B “possibly carcinogenic” viruses (HPV 26, 30, 34, 53, 66, 67, 69,70, 73, 82, 85, 97), those phylogenetically related to group 1 genotypes or presenting carcinogenic potential in mechanistic studies (4–9).

Genital HPV infections are highly prevalent among sexually active men and women worldwide (10). The IARC HPV prevalence survey carried out in 15 areas in four continents showed that oncogenic HPVs are also very common among cytological normal women with a variation of nearly 20 times in the HPV prevalence depending on age groups and geographic regions (11, 12). A large meta analysis, comprising more than one million women with normal cervical cytology, showed an overall HPV prevalence of 11.7% (95% confidence interval, 11.6–11.7). The highest HPV frequency rates were observed in Sub-Saharan Africa (24%), Eastern Europe (21.4%), and Latin America (16.1%) (13) paralleled by the highest incidence and mortality rates for cervical cancer.

Migrant women originating from low- and middle-income developing countries with limited resources in their health services and lack of cervical cytology screening programs have an increased risk of sexual transmitted diseases and HPV-related neoplasia (14–16). Furthermore, a study showed that the excess risk is only appreciable in women coming from high migration pressure countries with a high prevalence of HPV (17). In Italy at the end of 2011, there were 3536062 immigrants legally resident of which 32% from Eastern and Central Europe, 31% from Africa, and 25.6% from Asia^1^. The Eastern European countries are still experiencing high incidence of cervical cancer, lack of appropriate cervical cancer prevention programs, and paucity of HPV epidemiological surveys (13, 18, 19). Only a few studies have analyzed HPV genotype distribution in the former Soviet Union countries (20, 21). Tachezy et al. (22) recently reported an HPV prevalence above 28% in the screening population of Czech women with HPV16 as the most prevalent virus (19% of all HPV infections) (22). No HPV prevalence studies and analysis of specific genotypes have been performed in women residing in several other Eastern European countries.

The aim of the current study was to describe the prevalence of HPV infection and the distribution of oncogenic genotypes in migrant women living in Italy along with the prevalence of HPV-related cervical neoplasia. The analysis was based on the three prospective studies including migrant women enrolled in Apulia, Campania, and Sicily (23–25) and one retrospective study including cases of invasive cancer in Italian women and in those born abroad (26).

## Materials and Methods

### Study subjects and laboratory test

Published data on the analysis of HPV type distribution in migrant women living in Italy were searched in Medline using the terms [“HPV” OR (“human” AND “Papillomavirus”)] AND (“migrant” OR “immigrant” OR “foreign” OR “asylum”) AND (“Italy”). When data were published more than once, only the publication with the largest sample size was included. Additional relevant references cited in retrieved articles were also evaluated. For the studies including women originating from more than one geographic location, the data were divided into components for each continent. The review was limited to studies that (1) included the analysis of cytological samples or paraffin embedded cancer tissues obtained from migrant women living in Italy; (2) provided cytological/histological grading; and (3) used broad spectrum HPV–DNA-based PCR assays able to detect and genotype at least the 12 high-risk HPVs included in the group 1. The search was updated on 15 November 2013.

Four studies fulfilled the inclusion criteria. (1) The study by Chironna et al. (23) analyzed 150 migrant women, housed in the Asylum Seekers Center in Bari Palese (Apulia, South Italy). Among the 168 foreign women received and examined by the Center’s medical staff in the asylum during the period May to August 2010, 89% accepted to participate in the study. The women, after obtaining informed consent, underwent cervical cytological screening (Pap test) and HPV testing with the Linear Array HPV genotyping test (Roche Diagnostics, Milan, Italy) able to identify HPV genotypes 6, 11, 16, 18, 26, 31, 33, 35, 39, 40, 42, 45, 51, 52, 53, 54, 55, 56, 58, 59, 61, 62, 64, 66, 67, 68, 69, 70, 71, 72, 73, 81, 82, 83, 84, IS39 (82), and CP6108 (cand 89). (2) The study by Tornesello et al. (24) included 233 foreign women living in Campania region and enrolled at Pineta Grande Hospital from January through October 2004, and at the Federico II University from February 2007 through March 2010. HPV detection and genotyping were carried out using broad spectrum PCR with the modified general primers MGP followed by direct sequencing analysis/reverse line blot (RLB). The system was evaluated as proficient for detection of HPV 16, 18, 31, 33, 35, 39, 45, 52, 56, 58, 59, 66, and 68b^2^. (3) The study by Giovannelli et al. (25) included 115 migrant women who consecutively attended the outpatient clinic of Dipartimento di Medicina Clinica e delle Patologie Emergenti at the Policlinico University of Palermo between December 2007 and June 2008. All samples were tested using two systems for HPV identification and typing, the INNO-LiPA HPV Genotyping system (Innogenetics N.V., Ghent, Belgium), detecting 24 different HPV types and the wide-spectrum nested PCR assay with MY09/11 and GP05+/06 + primers followed by direct nucleotide sequencing analysis. (4) The pooled analysis of three studies evaluating the HPV prevalence in invasive cervical cancer in Italy by Giorgi Rossi et al. (26) included 526 Italian women and 48 women born abroad. In the three studies different PCR-based strategies were used for HPV detection and genotyping, however, the search for at least the 18 oncogenic and probably oncogenic HPV genotypes was achieved in each study (27–29).

### Statistical analysis

The frequency of each HPV genotype in cancer cases from Italian women (expected) was compared with the frequency of the corresponding genotype in cancers from born abroad women (observed). Ratios of observed and expected frequencies were calculated for each genotype and contingency tables were used to test for statistical significance with χ^2^ test, 1 degree of freedom.

## Results

A total of 499 cervical cytology samples and 46 cancer biopsies from foreign women living in Italy were included from the original studies (Table 1). The overall prevalence of HPV infection in foreign women was 51.1% (255 out of 499) among cytological specimens and 95.8% (46 out of 48) in cervical cancer. In Apulia region above 89% of women housed at the Asylum Seekers Center in Bari gave their consent to participate to the study. The mean age of enrolled women was 24 ± 4 years (range 13–41) and abnormal cytology was found in 4.4% (four ASCUS and one low SIL) of migrants with an overall HPV prevalence of 39.1%. The migrants enrolled in Campania region, self-referring for care to the gynecologic clinics, had a mean age of 37.6 ± 10.8 years (range 20–69) and 7.3% presented cervical cytological abnormalities (ASCUS *n* = 2; low SIL *n* = 12; high SIL *n* = 3). The prevalence rates of HPV infection were 57.9 and 94.1% among women with normal and abnormal cytology, respectively. In Sicily foreign women, enrolled among those self-referring for gynecologic care, the median age was 31.2 years (range 22–55) and the frequency of cases with abnormal cytology was 23.5% (27 out of 115). HPV prevalence was 42% (37 out of 88) and 66.7% (18 out of 27) in women with normal and abnormal cytology, respectively. Age-stratified HPV prevalence in the three regions showed a higher infection rate at younger ages with peaks of 53.1% among women ≤20 years old in Apulia and 70 and 68.3% in women aged 21–24 and 25–29, respectively, in Campania region, and 72.4% among women aged 22–27 in Sicily (Table 2). The prevalence declined to 33% in migrant women >30 years of age in Apulia and Sicily and remained above 55% in women enrolled in Campania region. Age-specific HPV distribution stratified by geographical origin showed in the 21–24 and 25–29 age groups a higher frequency of HPV infection in Southern and Eastern Europe women (71.4 and 78.6%, respectively) compared with Africa women (48.2 and 48.8%, respectively), Table 3. Among the 48 women affected by cervical cancer and born abroad, the mean age (45.9 ± 11.8, range 31–70) was significantly lower than that of Italian women with cervical cancer (mean age 52.0 ± 14.5, range 21–97) (*p* = 0.0049).

**Table 1:** **Regional distribution and overall HPV prevalence among 499 migrants enrolled in three Italian regions: Apulia, Campania, and Sicily**.

Region and country of origin	Normal cytology	Norm cytol HPV-pos	SIL^a^	SIL HPV-pos	Total	Total HPV-pos	Reference
	*N*	*N* (%)	*N*	*N* (%)	*N*	*N* (%)	
**SOUTHERN AND EASTERN EUROPE**							
Albania (*n* = 6), Byelorussia (*n* = 1), Bulgaria (*n* = 12), Macedonia (*n* = 2), Moldova (*n* = 1), Poland (*n* = 6), Romania (*n* = 11), Russia (*n* = 17), Serbia (*n* = 3), Ukraine (*n* = 95)	139	78 (56.1)	15	14 (93.3)	154	92 (59.7)	Tornesello et al. (24)
Eastern Europe (*n* = 55)					55	28 (50.9)	Giovannelli et al. (25)
**SOUTHERN AND CENTRAL AMERICA**							
Peru (*n* = 1), Ecuador (*n* = 1), Colombia (*n* = 2), Brazil (*n* = 2), Uruguay (*n* = 1), S. Domingo (*n* = 1)	8	5 (62.5)			8	5 (62.5)	Tornesello et al. (24)
**AFRICA**							
Tunisia (*n* = 2), Nigeria (*n* = 46), Morocco (*n* = 1), Ivory Coast (*n* = 2), Congo (*n* = 1), Benin (*n* = 1), Ethiopia (1), Mozambique (*n* = 1), Republic South Africa (*n* = 1)	54	30 (55.5)	2	2 (100)	56	32 (57.1)	Tornesello et al. (24)
Nigeria, Ivory Cost and Mali (*n* = 141); Somalia, Eritrea (*n* = 40)	109	39 (35.8)	5	4 (80)	151	59 (39.1)	Chironna et al. (23)
Sub-Saharan Africa (*n* = 60)					60	28 (46.7)	Giovannelli et al. (25)
**SOUTHERN ASIA**							
Sri Lanka (*n* = 8), Kyrgyzstan (*n* = 6), Philippines (*n* = 1)	15	11 (73.3)			15	11 (73.3)	Tornesello et al. (24)
Total	325	163 (50.1)	22	20 (90.9)	499	255 (51.1)	

*^a^SIL (squamous intraepithelial lesion) comprises all cytological abnormalities. ASCUS (atypical cells of undetermined significance, low grade SIL and high grade SIL)*.

**Table 2:** **Type-specific HPV prevalence among migrant women with or without cervical abnormalities enrolled in Apulia (*n* = 151), in Campania (*n* = 233), and in Sicily (*n* = 115) region**.

HPV type	Apulia	Campania	Sicily
	
	*N* (%)^a^		
HPV-neg	92 (60.9)	93 (39.9)	60 (52.2)
HPV-pos	59 (39.1)	140 (60.1)	55 (47.8)
HPV group 1-pos^b^	48 (31.2)	114 (48.9)	50 (43.5)
**HPV-POS BY AGE**			
≤20	17 (53.1)	2 (100)	
21–24	26 (41.3)	14 (70.0)	21 (72.4)^c^
25–29	12 (29.3)	28 (68.3)	15 (51.7)^d^
>30	4 (33.3)	96 (56.5)	19 (33.3)^e^
**HPV GROUP 1^b^**			
16	7 (11.9)	61 (43.6)	9 (16.4)
18	3 (5.1)	2 (1.4)	7 (12.7)
31	4 (6.8)	13 (9.3)	5 (9.1)
33	1 (1.7)	5 (3.6)	2 (3.6)
35	5 (8.5)	3 (2.1)	2 (3.6)
39	5 (8.5)	2 (1.4)	4 (7.3)
45	5 (8.5)	3 (2.1)	3 (5.5)
51	5 (8.5)	1 (0.7)	7 (12.7)
52	3 (5.1)	7 (5.0)	6 (10.9)
56	2 (3.4)	8 (5.7)	2 (3.6)
58	5 (8.5)	9 (6.4)	3 (5.5)
59	3 (5.1)		
**HPV GROUP 2–3**			
6	1 (1.7)	2 (1.4)	2 (3.6)
11			1 (1.8)
26	1 (1.7)		
32		1 (0.7)	
40	1 (1.7)		1 (1.8)
42	61 (0.2)	2 (1.4)	1 (1.8)
43			1 (1.8)
44		2 (1.4)	4 (7.3)
53	8 (13.6)	9 (6.4)	5 (9.1)
54	5 (8.5)	3 (2.1)	3 (5.5)
55	3 (5.1)		
61	6 (10.2)	1 (0.7)	
62	3 (5.1)	3 (2.1)	
64	1 (1.7)		
66	5 (8.5)	2 (1.4)	4 (7.3)
67	2 (3.4)	2 (1.4)	
68	5 (8.5)	2 (1.4)	5 (9.1)
70	3 (5.1)	8 (5.7)	2 (3.6)
72	2 (3.4)		
73	2 (3.4)	1 (0.7)	
74			3 (5.5)
81	6 (10.2)	2 (1.4)	2 (3.6)
82	5 (8.5)		
83	1 (1.7)	4 (2.9)	2 (3.6)
84	5 (8.5)		1 (1.8)
90		1 (0.7)	
cand89	3 (5.1)		3 (5.5)

*^a^Percentages of individual HPV genotypes are calculated over the total number of HPV-positive women in each geographical region. The sum of the different HPV types exceeds the total number of HR HPV-positive due to multiple infections*.

*^b^HPV classification is presented in accordance to the risk assessed by the IARC Monograph working group (6)*.

*^c^Women in the age range 22–27*.

*^d^Age range 27–32*.

*^e^Age range 33–55*.

**Table 3:** **HPV type-specific prevalence among migrant women from Africa (*n* = 207), Southern and Eastern Europe (*n* = 154), Southern and Central America (*n* = 8), and Southern Asia (*n* = 15)**.

	All migrants^a^	Africa	South and East Europe	South and Central America	Southern Asia
	
	*N* (%)				
HPV-neg	240 (48.1)	116 (56.0)	62 (40.3)	3 (37.5)	4 (26.7)
HPV-pos	259 (51.9)	91 (44.0)	92 (59.7)	5 (62.5)	11 (73.3)
HPV group 1-pos	196 (39.3)	69 (33.3)	77 (50.0)	4 (50.0)	10 (66.7)
**HPV-POS BY AGE^b^**					
≤20	19 (55.9)	18 (54.5)	1 (100)		
21–24	40 (48.2)	28 (41.2)	11 (78.6)	1 (100)	
25–29	40 (48.8)	21 (38.9)	15 (71.4)		4 (80.0)
>30	100 (54.9)	24 (49.0)	65 (55.1)	4 (80.0)	7 (70.0)
**GROUP 1**					
16	70 (27.0)	14 (15.4)	47 (51.1)	2 (40.0)	5 (45.5)
18	10 (3.9)	3 (3.3)		1 (20.0)	1 (9.1)
31	22 (8.5)	7 (7.7)	10 (10.9)		
33	7 (2.7)	4 (4.4)	1 (1.1)	1 (20.0)	
35	9 (3.5)	6 (6.6)	1 (1.1)	1 (20.0)	
39	11 (4.2)	5 (5.5)	2 (2.2)		
45	10 (3.9)	5 (5.5)	2 (2.2)		1 (9.1)
51	13 (5.0)	5 (5.5)	1 (1.1)		
52	14 (5.4)	4 (4.4)	4 (4.3)		1 (9.1)
56	11 (4.2)	4 (4.4)	5 (5.4)		1 (9.1)
58	16 (6.2)	9 (9.9)	4 (4.3)	1 (20.0)	1 (9.1)
59	3 (1.2)	3 (3.3)			
**GROUP 2–3**					
6	5 (1.9)	1 (1.1)	2 (2.2)		
11	1 (0.4)				
26	1 (0.4)	1 (1.1)			
32	1 (0.4)		1 (1.1)		
40	2 (0.8)	1 (1.1)			
42	8 (3.1)	6 (6.6)	1 (1.1)		1 (9.1)
43	1 (0.4)				
44	6 (2.3)	1 (1.1)	1 (1.1)		
53	22 (8.5)	11 (12.1)	6 (6.5)		
54	11 (4.2)	7 (7.7)	1 (1.1)		
55	3 (1.2)	3 (3.3)			
61	7 (2.7)	6 (6.6)	1 (1.1)		
62	5 (1.9)	3 (3.3)	2 (2.2)	1 (20.0)	
64	1 (0.4)	1 (1.1)			
66	11 (4.2)	5 (5.5)	2 (2.2)		
67	4 (1.5)	3 (3.3)	1 (1.1)		
68	12 (4.6)	5 (5.5)	2 (2.2)		
70	13 (5.0)	7 (7.7)	4 (4.3)		
72	2 (0.8)	2 (2.2)			
73	3 (1.2)	2 (2.2)	1 (1.1)		
74	3 (1.2)				
81	10 (3.9)	8 (8.8)			
82	5 (1.9)	5 (5.5)			
83	7 (2.7)	2 (2.2)	3 (3.3)		
84	6 (2.3)	5 (5.5)			
90	1 (0.4)			1 (1.1)	
cand89	6 (2.3)	3 (3.3)			

*^a^The group of all migrants includes cases from Africa and Southern and Eastern Europe not stratified by origin in the original article (25)*.

*^b^The HPV-positive group stratified by age do not include cases by Giovannelli et al. (25)*.

Thirty-nine different genotypes have been identified and among them the overall prevalence of group 1 HPVs (type 16, 18, 31, 33, 35, 39, 45, 51, 52, 56, 58, and 59), was 31.2, 48.9, and 43.5% in women from Apulia, Campania, and Sicily, respectively. The most common viral genotypes were HPV16 and HPV31 representing 27 and 8.5% of all infections, respectively. The relative frequencies of specific genotypes in migrant women from Apulia, Campania, and Sicily were quite comparable except for HPV59 representing 8.5% of infections in Apulia women and absent in other groups, and HPV16, highly prevalent in Campania (43.6% of all infections) compared to Apulia (11.9%) and Sicily (16.4%) migrant women (Table 2).

The frequency of HPV infection and distribution of specific genotypes among the 499 migrant women stratified by the geographical origin is shown in Table 3. HPV sequences were amplified in 51.9% (259 out of 499) of the samples with detection rates of 44% (91 out of 207) in Africa, 59.7% (92 out of 154) in Southern and Eastern Europe, 62.5% (5 out of 8) in Southern and Central America, and 73.3% (11 out of 15) in Southern Asia women. The overall prevalence of group 1 high-risk HPVs, including the fraction contributed by multiple infections, was 39.3% in all migrant women, 33.3% in women from Africa, 50% in those from Southern and Eastern Europe, 50% in those from Central America and 66.7% in women from Southern Asia (Table 3). As expected, HPV16 was the most prevalent genotype representing 15.4, 51.1, 40, and 45.5% of all infections in Africa, Southern and Eastern Europe, Southern and Central America, and Southern Asia, respectively. Other common viral types were HPV31 in Southern and Eastern European (10.9%) and HPV58 in African HPV-positive women (9.9%). Of note, the probable high-risk HPV53 was particularly frequent in African women representing 12.1% of all infections.

Infections with multiple HPV genotypes were very frequent being present in 45.7, 8.6, and 18.3% of HPV–DNA positive migrant women in Apulia, Campania, and Sicily, respectively.

The pooled analysis of three large studies on HPV prevalence in cervical cancer in Italy included 526 cases from women born in Italy and 48 cases from women born abroad. HPV sequences were detected in 95.8% of cases in both cancer groups (504 out of 526 in native Italian; 46 out of 48 in born abroad) and HPV16 was the most common genotype representing 67.5% (340 out of 504) and 71.7% (33 out of 46) of all infections in native Italian and born abroad women, respectively. The distribution of HPV genotypes did not differ significantly between Italian and born abroad women (Table 4), with the exception of an excess for HPV33 in immigrants with borderline statistical significance (*p* = 0.076).

**Table 4:** **HPV type-specific prevalence among cervical cancer cases in native Italian and abroad born women**.

	Born in Italy	Born abroad	Observed/expected	*p*-Value*
	
	*N* (%)^a^			
HPV-neg	22 (4.2)	2 (41.7)		
HPV-pos	504 (95.8)	46 (95.8)		
**GROUP 1**				
16	340 (67.5)	33 (71.7)	1.06	0.636
18	57 (11.3)	3 (6.5)	0.57	0.318
31	43 (8.5)	6 (13.0)	1.52	0.314
33	11 (2.2)	3 (6.5)	2.97	0.076
35	11 (2.2)	1 (2.2)	0.99	0.992
39	4 (0.8)	1 (2.2)	2.72	0.349
45	36 (7.1)	3 (6.5)	0.91	0.867
51	7 (1.4)		0	0.420
52	9 (1.2)		0	0.360
56	5 (1.0)	1 (6.5)	2.18	0.464
58	18 (3.6)	1 (6.5)	0.60	0.615
59	5 (1.0)	1 (6.5)	2.18	0.464
**GROUP 2–3**				
6	2 (0.4)		0	0.667
42	2 (0.4)		0	0.667
53	4 (0.8)		0	0.543
66	5 (1.0)		0	0.496
67	1 (0.2)		0	0.762
68	3 (0.6)		0	0.600
70	4 (0.8)		0	0.543
73	9 (1.8)		0	0.360
83	1 (0.2)		0	0.762

*^a^Percentages of individual HPV genotypes are calculated over the total number of HPV-positive women in each geographical region. The sum of the different HPV types exceeds the total number of HR HPV-positive due to multiple infections*.

*Probability of observed/expected ratio = 1, χ^2^.

## Discussion

The overall HPV prevalence of 51.9% observed among 499 migrant women living in Southern Italy regions such as Apulia, Campania, and Sicily is much higher compared to the infection frequencies of 13.4%, observed among 3,817 Italian women attending organized cervical cancer screening, and of 19.7% found in183 Italian women with normal cytology self-referring for gynecologic care (30, 31). Similar findings have been reported in migrant groups in Spain where the HPV prevalence among migrants from Colombia, Ecuador, or other Latin American countries was found three times higher than the 8.2% prevalence in Spanish-born women (32), and a peak of 61% in HPV prevalence was observed among sex workers from Eastern Europe (33).

The high burden of HPV infection in migrants may reflect the high viral prevalence in their country of origin. In fact all women enrolled in the Apulia Asylum Seekers center, immigrated mainly from Nigeria, Ivory Cost and Mali, were short-term immigrants enrolled soon after their arrival in Italy (23). Moreover, the nucleotide sequence analysis of HPV16, 52, and 58 amplimers from samples of African immigrant women showed nucleotide signatures distinctive of viral variants specifically distributed in the country of origin of these women (34).

A total of 39 viral genotypes were identified including all HPVs classified as carcinogenic to humans (group 1) (6). The relative frequencies of individual HPV genotypes are in agreement with the general distribution found in previous HPV surveys and meta analysis comprising women with normal cytology worldwide (11, 13, 35). Among HPV-positive migrant women, the fraction of HPV16 infections varied between 15.4% in Africa and 51.1% in Southern and Eastern Europe, confirming previous observations on the heterogeneous distribution of viral genotypes in different populations (11). Nevertheless, the majority of cervical cancer has been found positive for HPV16 in all world regions suggesting a strong long-term risk for CIN3 and invasive cancers following infection with HPV16 (35).

The high burden of HPV infection in migrants correlates very well with the increased risk of cervical cancer and CIN3 compared to Italian women. Several studies have documented a higher occurrence of infection-related cancer, including cervical cancer, in immigrants from low and medium-income developing countries compared to the native populations (36–41), often reflecting the incidence in their countries of origin (37, 41). Crocetti et al. (16) have reported an increased risk of invasive and *in situ* cervical cancer, during the years 2000–2004, among women aged 25–59 who were born in different countries but residing in central Italy. The incidence of invasive cervical cancer among women born in Central and South America and the Caribbean (60.5 per 100,000) and in Central and Eastern Europe (38.3 per 100,000) was statistically significant higher than that of Italian born women (6 per 100,000) (16, 42). Accordingly, Di Felice et al. (17) recently reported a higher risk of cervical cancer and CIN3 (SIR = 4.1, 95% CI = 2.2–6.9; SIR = 2.0, 95% CI 1.7–2.5, respectively) in women originating from high HPV prevalence countries and residing in the Reggio Emilia province (Northern Italy) in 2002–2009, compared to Italian women. On the other hand, there was no increased risk of cervical cancer in women coming from low HPV prevalence countries, suggesting that HPV prevalence in country of origin is an important determinant of cervical neoplasia incidence (17).

In Italy immigration is a relatively recent phenomenon: the proportion of immigrants was less than 0.6% in 1990, 2.3% in 2001 and reached the 6.8% in 2011^3^ (43). This means that most of immigrants arrived in our country less than 5 years ago. Therefore, the prevalence of HPV infections, particularly those clinically relevant, as well as the high risk of cervical cancer may reflect the figures of their origin countries.

Several studies have shown that the higher cancer incidence among migrant women living in Italy may be explained either by the higher HPV infection prevalence or the lower uptake of Pap test in the country of origin (44, 45). Notably, Di Felice et al. (17) reported a significant lower ratio of CIN3/cancer among women from high (ratio 2.6) and low (ratio 3.6) HPV prevalence countries than in Italian women (ratio 7.4), suggestive of a role of low screening uptake in cervical cancer incidence among immigrants (17). Cervical cancer screening programs in Italy are available for both regular and illegal migrants. Participation in the screenings is only slightly lower among migrants compared to Italians, 42.2 vs. 46.9% (46), but Pap test coverage is much lower, i.e., 69 vs. 78% (47) due to a minor contribution of spontaneous Pap testing in immigrants compared to Italians (17). Those regions with an incomplete activation of organized screening program are continuing and increasing those health inequalities that already affect immigrant women.

A limitation of the present pooled analysis includes a possible variation in the HPV genotype-specific sensitivity of different PCR assays used in the different studies. In fact, in the meta analysis including more than one million women with normal cytology, it was shown that the adjusted HPV prevalence for the MY09/11-based consensus PCR primers was significantly higher than that observed for GP5/6 or GP5+/6 + oligonucleotides, and that the PCR SPF10 primer system presented the better sensitivity and the highest HPV detection rate (13). The three PCR assays used to determine HPV prevalence among migrant women, namely INNO-LiPA HPV assay (25), Linear Array HPV test (23), and MY09/MY11 and MGP primer system (24), have been validated as proficient to detect the 12 oncogenic HPV genotypes in the HPV LabNet International Proficiency Study (48). Therefore, if some HPV genotypes have been underestimated in some study, they are not among those conferring cancer risk. A further limit is represented by the different enrollment strategies adopted in the different studies. In particular women enrolled in Campania and Sicily were among those self-referring to gynecological outpatient clinics for health assistance, and supposed to be more at risk for sexual disease; women enrolled in Apulia, instead, voluntary underwent an offered screening.

On the other hand, a strength of the present study was represented by the relatively large population sampled in three Italian regions receiving highest immigration flows from low- and middle-income countries.

## Conclusion

The high prevalence of oncogenic HPVs and cervical cancer risk among migrant women, together with the lower participation in screening programs, demands for an urgent implementation of preventive strategies to increase screening and vaccine coverage and viral monitoring of uncommon HPV genotypes potential spreading in settled population.

## Conflict of Interest Statement

Dr. Paolo Giorgi Rossi is Principal Investigator of a project sponsored by the Italian Ministry of Health (data owner) and is in contact for reagents at a reduced price or free of cost from Roche Diagnostics, Hologic Gen-Probe, Abbott, and Qiagen. The other authors declare that the research was conducted in the absence of any commercial or financial relationships that could be construed as a potential conflict of interest.

## Footnotes

^1^http://demo.istat.it/altridati/noncomunitari/index.html

^2^http://www.who.int

^3^http://demo.istat.it/
